# Evaluation of Recent Intranasal Drug Delivery Systems to the Central Nervous System

**DOI:** 10.3390/pharmaceutics14030629

**Published:** 2022-03-12

**Authors:** Tyler P. Crowe, Walter H. Hsu

**Affiliations:** 1 Carver College of Medicine, University of Iowa, Iowa City, IA 52242, USA; tyler-crowe@uiowa.edu; 2 Department of Biomedical Sciences, Iowa State University, Ames, IA 50011, USA

**Keywords:** intranasal, nose-to-brain, CNS, drug delivery, nanocarriers

## Abstract

Neurological diseases continue to increase in prevalence worldwide. Combined with the lack of modifiable risk factors or strongly efficacious therapies, these disorders pose a significant and growing burden on healthcare systems and societies. The development of neuroprotective or curative therapies is limited by a variety of factors, but none more than the highly selective blood-brain barrier. Intranasal administration can bypass this barrier completely and allow direct access to brain tissues, enabling a large number of potential new therapies ranging from bioactive peptides to stem cells. Current research indicates that merely administering simple solutions is inefficient and may limit therapeutic success. While many therapies can be delivered to some degree without carrier molecules or significant modification, a growing body of research has indicated several methods of improving the safety and efficacy of this administration route, such as nasal permeability enhancers, gelling agents, or nanocarrier formulations. This review shall discuss promising delivery systems and their role in expanding the clinical efficacy of this novel administration route. Optimization of intranasal administration will be crucial as novel therapies continue to be studied in clinical trials and approved to meet the growing demand for the treatment of patients with neurological diseases.

## 1. Introduction

Neurological diseases represent a significant and growing disease burden both in the U.S. and worldwide. Alzheimer’s Disease (AD) currently affects nearly 5 million Americans, incurring an annual estimated societal cost of >USD 100 billion [1,2]. This places AD among the most expensive diseases in the U.S., with regards to both the financial and human toll. This is projected to only increase, with prevalence climbing up to nearly 14 million Americans by 2050.

Despite this massive and growing problem, our treatments for AD and other neurological diseases remain incredibly limited, largely due to the anatomy of the central nervous system (CNS) and the blood-brain barrier (BBB). The BBB helps maintain homeostasis by severely limiting access to the CNS compartment through a combination of endothelial cells, intercellular tight junctions, and transport proteins [3,4]. Though lipophilic molecules can still access the CNS via diffusion, the movement of hydrophilic molecules across the BBB is reduced by 98–100% [5]. This is shown in many treatments for neurodegenerative diseases, such as levodopa for Parkinson’s Disease (PD), where <5% of the dose reaches the CNS. Low bioavailability in the CNS requires the use of larger doses, leading to increased adverse effects. Therefore, formulations which can improve CNS bioavailability will be increasingly important for medications to be effective.

Intranasal delivery directly to the CNS offers exciting potential to bypass the highly selective BBB and deliver a greater variety of therapeutic agents to the brain in greater concentrations. Intranasally administered bioactive peptides, e.g., insulin, glial-derived neurotrophic factor, or leptin have been shown to be delivered directly from the nose to the brain in rodent models [6]. Though not every study has included the endpoint, many have shown a response in the animal such as improved cognition with insulin or decreased feeding with leptin. These thrilling animal model data have not been replicated in humans, however. Although several studies have demonstrated intranasal delivery to the brain like the animal models, it appears that a relatively small fraction of the dose is reaching the CNS [7,8]. Recent studies, such as intranasal oxytocin for autism, have failed to replicate the effects in humans [9]. Many of these studies were simply using a saline solution to administer the drug to the nasal cavity, just like in the animal models. It is becoming more apparent that due to anatomic and physiological differences between rodents and humans, more optimization is needed for the nose-to-brain pathway to reach its full therapeutic potential.

The purpose of this review is to discuss the various formulations, additives, and devices being studied to improve intranasal delivery to the CNS and the evidence for their potential.

## 2. Pathways to the CNS and Advantages of Intranasal Drug Delivery

### 2.1. Nasal Cavity Anatomy and Histology

**Figure 1:**
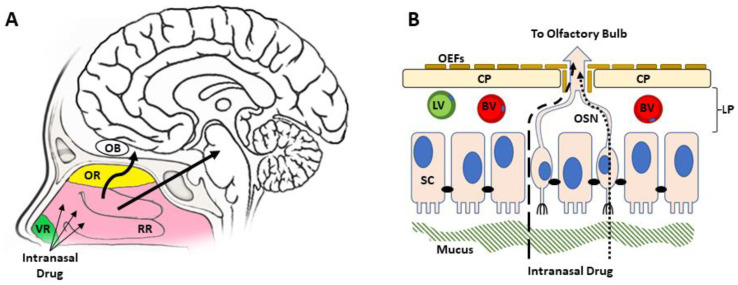
Anatomy and histology of the nasal cavity, epithelium, and transport pathway to the CNS. (**A**) Drugs administered to the nasal cavity cross the epithelium in either the superior olfactory region (OR) and move along the olfactory nerve (left arrow) to the olfactory bulb (OB), or the lateral respiratory regions (RR) and the trigeminal nerve (right arrow) to the pons. (**B**) From the lamina propria (LP), drugs are transported to the CNS along the olfactory sensory neuron (OSN, left arrow) p through the cribriform place (CP). A similar process occurs along the trigeminal nerve. Drugs can also be lost to systemic absorption via lymphatics (LV) or vasculature (BV). The anterior vestibular region (VR) is minimally involved in the intranasal route to the brain.

The nasal cavity presents the most cephalic portion of the respiratory system, and the normal functions are to condition air for the respiratory system and facilitate olfaction [10,11]. The most anterior portion of the cavity vestibular region is characterized by a large amount of hair and mucus production, as well as a robust squamous epithelial lining [12,13]. It emphasizes this region’s role in protection from mechanical irritation, rather than secretory or sensory which is in the other regions of the cavity (Figure 1).

The cavity is bounded by the nasal floor (continuous with the roof of the mouth) below both the maxillary and ethmoid bones laterally. The conchae are found on the lateral wall and lined in respiratory epithelia, allowing them to play their role in filtering and humidifying inhaled air. This is collectively the respiratory region, and is lined with a single layer of pseudostratified, ciliated columnar epithelial cell also containing goblet cells. This allows for mucus production and removal, protecting the upper airway from inhaled irritants or dry air [14].

The most superior aspect of the nasal cavity is lined by olfactory epithelia, a pseudostratified layer of cells. Contrary to the respiratory epithelia of the rest of the cavity, olfactory epithelia contain olfactory neurons and Bowman’s glands. Unlike the mucus-secreting and protective Goblet cells, this function is to wash away odor molecules from the nearby neurons. Deep and superior to this is the cribriform plate of the ethmoid bone, through which the olfactory neurons will project to the olfactory bulb and the rest of the CNS.

### 2.2. Nasal Cavity Vasculature and Innervation

The nasal cavity has a rich vascular supply full of anastomosis that is mostly centered in areas lined with respiratory epithelia on both the lateral walls and septum. Blood is supplied by branches of both the internal and external carotid arteries, including the anterior and posterior ethmoid arteries, the sphenopalatine, and greater palatine arteries. Small regions are supplied by the superior labial branch of the facial artery as well. Blood is returned via the facial vein for the anterior portions of the cavity and via the maxillary or sphenopalatine veins posteriorly into the pterygoid plexus. Lymphatics drain both anteriorly and posteriorly to the submandibular nodes.

The nasal cavity is innervated by the olfactory nerve (CN I) and trigeminal nerve (CN V). The olfactory nerve is found in the superior, olfactory region of the cavity and is comprised of bipolar neurons projecting through both the surrounding epithelia and cribriform plate. These axons synapse on the olfactory bulb in the ventral forebrain. These neurons and the spaces surrounding them are the primary route of intranasal transport to the CNS, as discussed in greater detail below. The trigeminal nerve innervates the larger remainder of the cavity via its ophthalmic (V1) and maxillary (V2) branches. General sensation is the primary function of these portions of the trigeminal nerve; the maxillary (V2) branch also contains parasympathetic fibers from the facial nerve (CN VII, greater petrosal) which controls glandular secretions in the cavity, as well as postganglionic sympathetic fibers.

**Figure 2:**
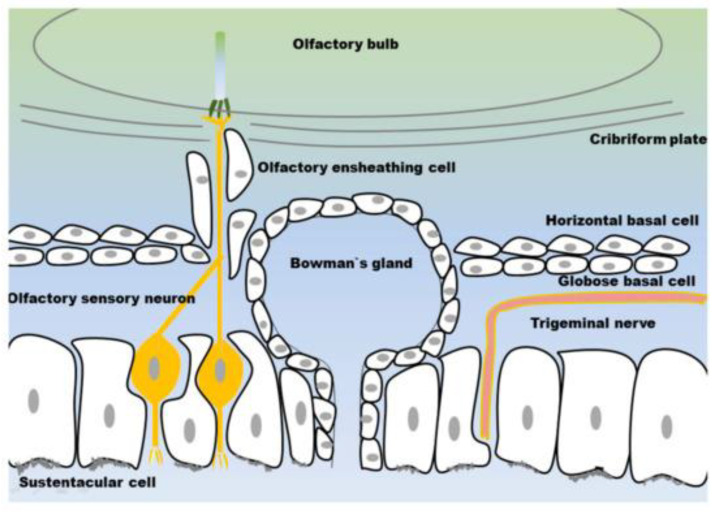
Comparison of olfactory (**left**) and respiratory epithelia (**right**), including location of the neurons within the sustentacular cell layers. The olfactory sensory neuron’s exposure to the nasal cavity helps explain the olfactory nerve’s larger role in intranasal delivery. Reprinted with permission from ref [15]. 2018 Stella Gänger.

Both the olfactory and trigeminal neurons are surrounded by pseudostratified epithelia in their respective regions of the nasal cavity. The trigeminal neuronal endings are only found within the lower regions of the epithelia, meaning they are not directly exposed to the nasal cavity. In stark contrast, for the olfactory neurons, cell bodies are within the epithelia and their cilia reach directly into the nasal cavity (Figure 2). This small difference is crucial for explaining why the smaller olfactory nerve plays a much larger role in intranasal transport, as detailed below. This point cannot be emphasized enough when considering how the histology ultimately informs the mechanism of intranasal delivery.

### 2.3. Mechanisms of Intranasal Transport to CNS

Understanding the various delivery systems used in intranasal-to-CNS therapies first requires a knowledge of the various routes and their respective mechanisms, since they dictate all factors from formulation and drug selection to safety and efficacy. Both the olfactory and trigeminal nerves have been shown to transport intranasally administered compounds to the CNS, but the olfactory nerve has been more thoroughly described in the literature. Recalling the different epithelia and vasculatures surrounding the nerves, the olfactory nerve provides better absorption and CNS transport with less systemic absorption. Additionally, due to the markedly shorter length of the nerve itself, the olfactory nerve is a significantly faster nerve than the trigeminal nerve. For the purposes of this article, the olfactory nerve will be discussed unless otherwise specified. 

**Figure 3:**
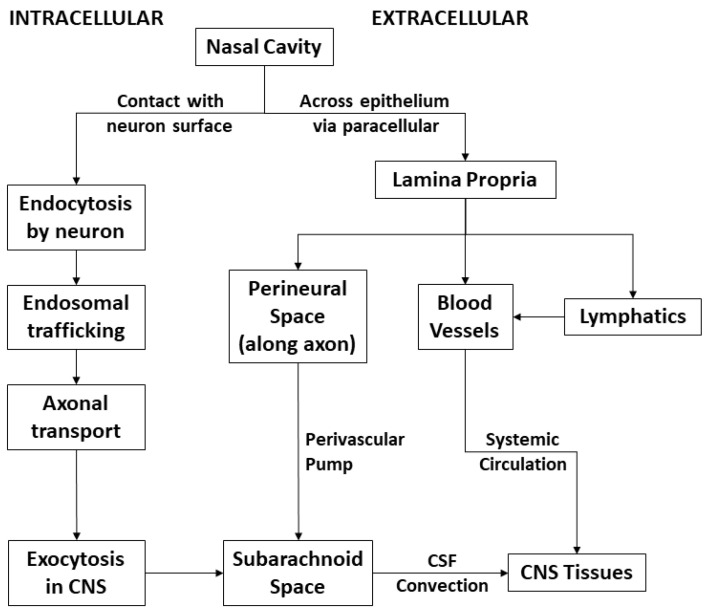
Schematic of intracellular and extracellular pathways for intranasal drug delivery.

Several thorough and high-quality reviews are available which detail the exact mechanisms by which intranasal administered drugs reach the brain [6]. Broadly, routes can be considered either intracellular or extracellular with respect to the neuron and each contains several mechanisms. Most molecules are transported via a combination of mechanisms (Figure 3).

#### 2.3.1. Intracellular Transport Mechanism

The intracellular mechanism of intranasal transport involves internalization of the drug by the neuron at the site of the epithelium, transport along the axon, and exocytosis at the other end within the CNS. Intracellular transport of intranasally administered drugs or therapies to the CNS begins via endocytosis of the administered agent by olfactory (or trigeminal) neurons. This can occur via non-specific or receptor-mediated endocytosis, though the existing literature appears to indicate that non-specific binding and uptake is far more common [6]. Now bound within an endosome, the substance undergoes trafficking via the Golgi network and axonal transport to reach the synapse. This is either the olfactory bulb for the olfactory nerve, or within the pons for the trigeminal nerve. Intracellular trafficking rate is independent of size and takes 0.74–2.67 h or 3.69–13.33 h for the olfactory and trigeminal nerves, respectively [16]. Once exocytosed, the agent is moved around the CNS, via either reuptake or convectional transport. The intracellular transport occurs only across non-neuronal epithelial cells, transporting the compound from the nasal cavity to the lamina propria. This is referred to as transcellular transport and requires subsequent transportation to reach the CNS.

#### 2.3.2. Extracellular Transport Mechanism

Extracellular transport can occur via a variety of mechanisms, which all share the basic principle of the drug moving through fluid in the spaces along which the neurons run. Notably this does not require binding and endocytosis by the neuron itself. First, the drug must cross the nasal epithelia from the nasal cavity. Although there are many tight junctions (TJs) between the epithelial cells, transient opening of the channels allows for the movement of molecules into the lamina propria. There are numerous methods of modifying the opening of TJs, which will be discussed in depth below. Additionally, olfactory neurons are not permanent like other neurons in the CNS, and they turnover every 30–60 days [17,18]. Between undergoing apoptosis and eventual replacement, this leaves a large opening among the surrounding sustentacular cells of the epithelium, which allows therapies access to the lamina propria.

From the lamina propria, intranasally administered drugs can be translocated to the brain via the perineural space. As the neurons which make up cranial nerves exit the CNS into the periphery, they take the layers of the mater ensheathing the nerve bundles [19]. This forms a perineural space with olfactory ensheathing fibroblasts (OEF) around the nerve filled with cerebrospinal fluid (CSF) that connects the subarachnoid space to the lamina propria. It is thought that drugs diffuse by bulk flow, pulsatile pressures created by concurrent arterioles, or to a lesser degree Brownian movement, to migrate into the CNS.

#### 2.3.3. Kinetic Evidence for Mechanisms

Based on limited evidence in murine models, intranasally administered drugs reach the CNS as early as 5 min post-administration, and more distal regions of the brain by 30 min [20,21]. Peak concentrations of intranasally administered compounds vary by region of the brain. The olfactory bulb peaks as soon as 10 min post-administration, while deeper regions such as the striatum take up to 30 min. The most distal locations such as the midbrain or hypothalamus require 30 min to reach the peak concentration post-administration [19]. The average peak time for the whole brain has ranged from 30 min to 2 h, depending on the study [20,22]. Since this evidence is from different tracer molecules, formulations, and model organisms, it is difficult to extrapolate these values for clinical considerations in humans. Lastly, clearance from the CNS is completed by ~4 h, giving an early indication of duration of effect for intranasally administered therapies.

Taken together, this evidence indicates that the majority of transport to the CNS occurs via the extracellular pathway and should be the focus of optimization. Axonal transport alone via the intracellular pathway would take 0.74–2.67 h for the olfactory nerve and 3.69–13.33 h for the trigeminal nerve, based on studies of neuronal axonal transport rate [16,23]. This is without the complexities of internalization and endosomal trafficking. Simple diffusion is not too different, 0.73–2.3 h and 17–56 h for the olfactory and trigeminal nerves, respectively. Only the extracellular pathway in combination with the pulsatile movements of arteriole provides congruent transport times seen above [23,24]. As the arterioles expand in systole, they compress the fluid in the surrounding sheath and create a wave which moves at a rate of 214 µm/min in in vitro studies. This “perivascular pump” is a very efficient mode of transport, translating to 0.33 h and 1.7 h for the olfactory and trigeminal nerves, respectively. Though even these fall short of the in vivo evidence in the literature, it is reasonable to think the absence of skull bone around the channels allows for greater energy dissipation and slowing of the pathway in vitro. Still, this extracellular pathway powered by systolic pulsations is the most plausible mechanism with the in vivo and in vitro radiotracer evidence, and thus should be the primary consideration for therapeutic design.

### 2.4. Distribution within the CNS Compartment

Understanding the distribution within the CNS of intranasally administered therapies is crucial for the ability to produce effective, targeted interventions with minimal off-target effects. Although there can be distribution within the tissues of the brain via continued intracellular transport, this is likely not the primary mechanism based on kinetic evidence and the known inefficiencies of non-specific endocytosis at synapses. Instead, CNS-wide distribution occurs via a combination of the convective bulk flow and perivascular pump discussed above. This is supported by evidence in rodents which shows cardiac output is positively correlated to rate of distribution, providing intranasally-administered compounds reach regions of the brain adjacent to the origins of the olfactory and trigeminal nerves within 20 min of administration, including the olfactory bulb, striatum, and brainstem [25,26,27,28]. Other structures in the cortex of the forebrain and midbrain peak afterward. Discrete pathways are still unclear, though evidence in rodents shows the rostral migratory stream (RMS) is crucial for distribution beyond the olfactory bulb [29,30], where resection of the RMS reduces distribution by over 80%. The importance of the RMS in humans is unclear, as the development of the RMS or analogous structures is not well supported in the literature. Further research is needed to help elucidate pathways for targeting brain tissues, though it is clear that at least some portions of intranasal therapies reach all regions of the brain in some capacity.

The current evidence in the literature indicates that targeting drugs to sites of action within the brain is a problem that will require further attention. Nonetheless, the advantages are clear. The olfactory bulb, pons, and adjacent structures have been demonstrated to receive a markedly high dose of drug when administered intranasally, compared to intravenous (IV) administration which showed preference for the choroid and adjacent structure [26,31,32,33]. Furthermore, bypassing the BBB allows for a more expansive range of drug or therapy profiles, which will be further discussed ahead.

## 3. Factors Affecting Intranasal Drug Delivery

Understanding the anatomy of the nasal cavity, the extracellularly-based transport pathway along the cranial nerves, and how drugs will reach the target tissues of the brain is crucial when considering the factors salient to effective intranasal delivery (Table 1). We will now look at those factors more closely and within a more clinically practical context. Optimization of these factors will be absolutely crucial to the development of an effective therapy in humans. After all, almost all the evidence discussed so far comes from rodent model organisms using trained professionals to carefully and precisely administer the drug. Human anatomy is not a one-to-one comparison with rodents, and our healthcare system does not have this luxury for administration of widespread, frequently dosed therapies; especially in patients with limited transportation due to neurological decline.

### 3.1. Mucus

The first barrier any therapy will encounter is the mucus coating which protects the nasal epithelium beneath. Mucus is a gel-like compound composed primarily of mucins which are mostly bound to membranes in mammals [34,35,36,37]. In addition to physically protecting the epithelium from the dry, harsh air moving through the cavity, mucus contains other substances with antimicrobial and immunomodulatory effects. There are a variety of mucin types in the whole family, and these tend to vary in proportion between organisms and disease states.

Mucin uses a strongly negative net charge to dry in water when forming a gel. While this is neutralized somewhat by the presence of cations e.g., Ca^2+^ and H^+^, this charge must be considered for formulation. Hydrophobic and charged hydrophilic molecules have been shown to diffuse poorly through mucus, whereas uncharged hydrophilic molecules are able to diffuse rapidly through the mesh of mucins nearly the speed of water for smaller molecules [65,66,67,68,69]. Drugs larger than 500 Da in size will be especially prone to poor mucus diffusion and becoming stuck, though most drugs will be smaller than 500 Da in size, thus it is not an important issue [15,70]. Additionally, the thickness of mucus can vary greatly depending on water content. Nasal mucus is one of the thinnest mucus types in the body; therefore, this is likely not a significant formulary consideration in most clinical cases [15]. Lastly, the rate of turnover of mucus (see below) must be considered. It appears that the addition of mucoadhesive coatings can increase absorption. Though this addition can be useful for increasing bioavailability, it may be limited since the nasal cavity produces a tremendous volume of mucus (20–40 mL per day) which is quickly turned over by ciliary propulsion (every 10–20 min) [38]. Even this rate varies in individuals’ nasal passages, as the left and right passages alternate degrees of congestion throughout the day as a part of the well-described nasal cycle [71,72,73]. The olfactory epithelium lacks the motile cilia responsible for this movement, thus the rate of turnover is slower in the primary region of interest for intranasal nose-to-brain transport. However, an increase in expression of P-glycoprotein (P-gp) pumps in olfactory epithelia may negate this effect [74]. More research in this area will be required in the future to ultimately increase mucus permeation by intranasally administered therapies.

### 3.2. The Nasal Cavity Epithelium and Tight Junctions

Any intranasally administered drug must bind or cross the epithelial lining to reach the lamina propria before it can be transported further into the CNS. Recalling the mucus coating, presence of TJs and limited proportion of the total cavity this covers, optimizing a formulation to maximize crossing into the lamina propria will be crucial for any therapy. This is especially true since the lamina propria also drains fluids back into either systemic circulation, local glands for excretion, or via lymphatics to the deep cervical chain of lymph nodes. It is actually a relatively small fraction which will be carried along the nerves and to the parenchyma of the brain, thus increasing the total amount arriving to the lamina propria is crucial for clinical efficacy.

TJs are a protein complex made of occludins, claudins, and more that connect epithelial cells at the apical surface and typically separate the basolateral sides of cells from the lumen or cavity. TJs can be modulated to increase or decrease permeability across the membrane primarily through phosphorylation signaling pathways on occludins. Several compounds have been used to transiently decrease nasal epithelial TJ tightness and increase intranasal delivery amounts, including papaverine, poly-L-arginine, 12-O-tetradecanotlophorbol-13-acetate (TPA), and bisindolylmaleimide [39,40,41,42,43,44]. Broadly, these compounds either directly dephosphorylate TJs or inhibit the function of various kinases (especially protein kinase C to reduce the function of the proteins and increase membrane permeability, ranging from two- to four-fold. Other options such as chitosan, a chitin derivative, have been shown to increase epithelial permeability by affecting TJs. When formulated as a cationic coating for nanostructured lipid carriers, researchers have observed increased delivery across a membrane and stronger pharmacological response [75,76,77]. Given the constrictions imposed by mucus on the types of drugs, this can provide a broad range of drugs access to this administration route.

Modulation of TJs may not even be absolutely required for effective intranasal delivery. Olfactory sensory neurons (OSNs), the functional unit of the olfactory nerve that binds to molecules to transduce the sense of smell, are relatively short lived by neuronal standards and turnover every 30–60 days [18]. New OSNs actually grow into the same spots in the olfactory epithelia, meaning there are cell-sized holes in the membrane at any given time. Since compounds as small as insulin (5.8 kDa) and as large as albumin (65 kDa) have been successfully delivered to the CNS intranasally in a simple saline solution, crossing these passages (a process known as persorption) may provide a floor for amounts delivered, even if most of the paracellular spaces are closed off by TJs [20,78,79,80]. It should be noted that this is only true for OSNs; the trigeminal nerve endings terminate in the transcellular space of the epithelia and do not reach into the nasal cavity.

### 3.3. Size and Charge Matters

Finally, the very biochemical nature of the drug itself has an impact on intranasal delivery bioavailability. Small, uncharged, hydrophilic molecules can move most freely through mucus and the matrix of mucins. For example, a small molecule such as dopamine (DA, 0.15 kDa) has a five-fold increase in CNS concentrations compared to the much larger nerve growth factor (NGF, 26.5 kDa) when administered at the same concentration [45,46]. Generally, 0.4 kDa is considered small enough to freely diffuse and pass through the nasal epithelia; it is only over 1 kDa that a drop off in diffusion is seen. This size limit is not entirely inhibiting though, as molecules as large as wheat germ agglutinin–horseradish peroxidase (80 kDa) and even whole stem cells have been transported to some degree [47,48,49,50,51,52,53,54,55,56,57].

Nonpolar compounds are thought to be transported poorly to the CNS intranasally, though there is a growly body of evidence that the proper microemulsion formulation can greatly increase the intranasal brain area under curve (AUC) compared to IV administration of the compound. Indeed, there is evidence that with some drugs *increasing* the hydrophobicity can increase delivery to the CNS [58,59]. It is known that hydrophobic compounds cross biological membranes such as the nasal epithelia, blood vessels, or BBB well. This shows that not only are hydrophobic drugs capable of being administered intranasally with the correct formulation, but this may be an advantage.

Similarly, nanocarriers and emulsions can be used to help increase the efficient delivery of highly charged compounds. Though there is existing evidence that strong cations such as Mn^2+^ and Co^2+^ or charged proteins and small molecules can be delivered without special formulation [60], achieving a desired therapeutic effect will likely require nanocarrier utilization, as chronic administration may lead to irritation and discomfort in human patients. Since many neurological diseases are chronic and without curative therapies currently, tolerance to preparations with nanocarriers is of the utmost concern.

### 3.4. Brief Comparative Anatomy and Translational Limits

When considering all of the evidence reviewed thus far, as well as that below, it is important to distinguish between research conducted on humans and that conducted on animal models. Both the conditions of the laboratory, with its highly trained workforce and controlled environment, and the anatomical differences between species play a significant role in the generalizability of the data. Often, researchers are administering doses as low as 25 µL but usually closer to 200 µL in size in these experiments; a size selected because this is the maximum volume of the nasal cavity in the model rodents [61,62]. In humans, the nasal cavity is 6–7 mL in volume, which is impractical at best [63]. Furthermore, 50% of the rodent nasal cavity is covered in olfactory epithelia, compared to <5% in humans [81]. This limitation in area will make delivery to the CNS less efficient and adds emphasis on making sure administered drugs reach the correct region of the nasal cavity. Animals are also positioned at a 90-degree angle or on their back, which can be difficult for elderly patients with limited mobility if dosing multiple times a day. Lastly, animals are typically anesthetized in these studies for administration, which slows the respiratory rate and drug clearance, leading to an increase in absorption which would not be seen in fully conscious patients. Evidence of this is limited and unclear, however, as few studies included unanesthetized control subjects/groups for comparison [82].

## 4. Types of Intranasal Strategy for Brain Drug Delivery

Strategies to improve intranasal delivery to the CNS include additives to the formulation, nanocarriers or particles which allow for molecules to cross the membrane (such as lipophilic compounds), or devices that increase the amount of drug that reaches the upper olfactory region of the cavity (Table 2). Each strategy has its own advantages and disadvantages. As this therapy transitions from trained professionals using model organisms in laboratories to everyday patients (many with a neurological disease), a combination of strategies will likely be required for therapeutic success. Based on the factors and limitations discussed before, it is seen in that <1% of intranasal administered compounds typically reach the brain [81]. To avoid irritation of the nasal epithelia, there will be a maximum tolerable dose, so additional strategies and preparations will be required.

## 5. Preparation and Evaluation of Intranasal Drug Delivery Systems

### 5.1. Solutions Alone

Though likely inadequate for clinically efficacious use, there is mixed evidence for intranasal administration of a drug in saline or phosphate-buffered saline (PBS) alone, which warrants discussion. Some studies in rodents have reported increased nose-to-brain delivery in these simple solutions, such as 5-fjuorouracil (104% increase compared to IV administration of the drug), remoxipride (50% increase in brain/plasma AUC, or morphine (30-fold higher brain/plasma AUC compared to IV administration of the drug) [83,84,85]. Still, others have found no difference between intranasal and IV administrations, such as a study using a 5-HT_1A_ receptor antagonist UH-301 in rats [86]. This variance in delivery may ultimately be a product of the chemical natures of the specific drugs, but this emphasizes the need for optimized formulations. Nonetheless, these results can be viewed as further proof of concept, as even the least effective intranasal formulation can deliver more to the CNS than the IV route. Thankfully, there is a broad range of types and specific formulations to overcome this phenomenon, which will be discussed below.

### 5.2. Additives to Increase Nasal Barrier Permeability

Though many studies involve administering a compound in a simple saline solution or even just water, the known poor delivery of these formulations (<1% of total dose reaching the CNS) will necessitate the addition of substances that increase intranasal absorption. In principle, most of these additives work to increase the amount of drug crossing the nasal epithelia into the lamina propria. Though this does not specifically work to increase the fraction moving along the nerves into the CNS from the lamina propria, it can improve the AUC in the brain and reduce the amount of dose that simply exits the nasal cavity or is degraded within mucus.

Permeability enhancers can be defined as any substance which increases the permeability of the nasal epithelial or membrane diffusion. This can take the form of additions which allowed for greater diffusion across membranes, e.g., surfactants, lipids, and cyclodextrans [58,87]. These permeability enhancers are especially useful for the transport of hydrophilic compounds or macromolecules. A significant disadvantage of these agents is that the mechanism involves disruption of the nasal epithelia, which can lead to potentially toxic irritation of the mucosae with time [88]. Such adverse reactions would greatly reduce the clinical potential for any drug requiring repeated dosing. Some agents, e.g., dextran, sodium hyaluronate, and Cremophor RH40 appear to be non-irritating and non-toxic. This list is far from comprehensive [89].

Another method to increase nasal membrane permeability is to modulate the function of TJs, which can be done via chitosan [90]. Indeed, early evidence shows administration of N-cyclopentyladenosine with chitosan microparticles resulted in a 10-fold increase in brain concentration following intranasal administration, compared to administration of N-cyclopentyladenosine with mannitol-lecithin [131]. Even transient opening of the TJs allows a larger and more hydrophilic drug to pass more readily through the paracellular space and to the lamina propria. Chitosans are also mucoadhesives allowing for more drug to be held in the nasal cavity adjacent to the membrane, resulting in increased retention time and absorption. Chitosans are also well-characterized and considered to be safe, non-irritating, and biodegradable; a strong perk for a chronically administered therapy [132].

Modulation or addition of enzymes has been used to increase permeability and intranasal delivery to the CNS. Several studies have shown that additions of matrix metalloproteinases (MMPs) can increase the intranasal delivery of compounds. Several studies have found fluorescently labelled dextran (10 kDa) to only reach the CNS when co-administered with an MMP [28,133]. Another study found that the addition of an MMP doubled the amount of biologically-active enzyme chloramphenicol acyltransferase (75 kDa) intranasally delivered to the brain [134]. Both authors acknowledged that destruction of the nasal extracellular matrix will likely be irritating with time, which greatly limits the application of this formulation in practice. More research will be needed to clarify the long-term safety of MMPs. Another example of enzymatic-focused options would be to block epithelial P-gp activity. Though not all drugs are P-gp substrates. The high expression of P-gp in the BBB, nasal membranes, and olfactory bulb will greatly limit the transport of drugs which are P-gp substrates. Several studies have shown that transport of verapamil, a P-gp substrate, to the brain can be increased by either the addition of a P-gp inhibitor, e.g., rifampin or cyclosporin A, or the use of P-gp-deficient mice [74,91,92,93]. For drugs which are substrates for P-gp, this evidence is very encouraging.

It is important to remember these additives must be tailored to specific medications. In some instances, these formulations can actually decrease the amount of drug transported to the brain. In one study, a chitosan nanoemulsion decreased the amount of pralidoxime delivered to the brain beyond the olfactory bulb compared to administration in a saline solution [135]. The authors thought this was due to loading efficiency issues. While this is an exception rather than the rule, it serves as a reminder that more is not always better.

### 5.3. Other Additives

Vasoconstrictors are other co-administered compounds which have been shown to significantly increase intranasal transport to the brain. One study used a vasoconstrictor phenylephrine and found that it increased the brain/plasma AUC ratio for several neuropeptides [64]. By reducing the vascular supply to the mucosa, it seems less drug in the lamina propria is lost via venous or lymphatic return to systemic circulation, allowing for more drug to reach the brain. Since the perivascular pump is a potentially significant contributor to the movement of drugs along the axons, modulation of the vascular system may decrease transport along both the trigeminal and olfactory nerves as well. Further research will be required for the mechanism of intranasal delivery to be fully understood. However, for drugs particularly prone to absorption into the systemic circulation, the use of a vasoconstrictor remains an option.

Inhibition of enzymes has also been shown to increase intranasal delivery. The nasal cavity possesses numerous enzymes capable of metabolizing drugs. This protective feature can greatly limit the intranasal pathway for drugs which are metabolized by these enzymes. Several studies have shown that inhibition of proteases or cytochrome P-450 enzyme in the nasal mucosa increases the amount of drug transported from the nasal cavity to the brain [81,94,95]. This same principle applies to the brain as well. Acetazolamide is a carbonic anhydrase inhibitor which decreases CSF production in the brain. Pretreatment with acetazolamide has shown to increase CSF concentration of intranasal drugs in several studies [83,96]. For drugs which require CSF convection to distribute to their site of action in the CNS, this presents an interesting option. It should be noted that this effect is only seen in pretreatment and not co-administration.

Though much of the evidence is preliminary and in rodent models, there are several promising additives which can potentially improve intranasal delivery to a level sufficient for clinical applications without causing adverse reactions that would exclude clinical use. Due to the design of these studies, few have the health of the animals’ respiratory system as a measured endpoint. This will need to change before any of them can be thoroughly studied in humans. For that reason, chitosan in particular seems promising with its robust body of evidence and well-characterized safety profile, as do the other non-irritating or non-toxic permeability enhancers. However, intranasal delivery to the brain can be improved by altering the drug, not just the nasal mucosa.

### 5.4. Coatings

One major limitation of intranasal drug delivery is the mucus coating and its high rate of turnover due to the clearance by cilia, as described above. Several strategies have shown promise for improving the specific changes that muco-ciliary clearance poses. For large-size drugs which are significantly more prone to becoming stuck in the mucus, this is especially promising.

Mucoadhesives serve to improve the first step in intranasal transport by better adhering a drug to the mucus, allowing it to be absorbed. There is a broad category of generally positively-charged molecules, e.g., chitosan (and several derivatives), carboxymethylcellulose, polacrylic acid, etc. [15]. Functionally, they work by increasing the residence time of the drug to increase absorption. Since the olfactory region’s cilia are non-motile, mucoadhesives may be effective for drugs especially targeting the olfactory nerve over the trigeminal nerve. The evidence for this method is mixed, with studies finding no significant difference in the clearance of small peptides [97]. However, this may not be the case with all types of drugs. Other research has shown that the brain AUC for buspirone in a mucoadhesive formulation was 2.5 times greater than a simple saline intranasal or IV formulation [98]. Similar results have been seen in other studies that lacked proper administration controls, thus comparison of results is difficult [86,98,99]. This strategy may be limited to certain drugs which have uniquely high clearance, such as buspirone (0.4 kDa).

Ciliostatics complement mucoadhesives by slowing the clearance time of mucus, further increasing the residence time of intranasal drugs. There is a long list of both reversible and irreversible ciliostatics and ciliotoxic drugs. Chlorbutol and several hydroxybenzoates are examples of reversible drugs, while chlorocresol edetate, phenylmercuric acetate, and thiomersal are irreversible examples [15]. Even chitosan has shown potential as a ciliostatics. This is far from an exhaustive list, there is limited but long-standing evidence that the irreversible benzalkonium chloride does not result in morphological changes to the nasal mucosa or the effective mucus clearance of the cavity [100]. Though this was used as a treatment for allergic rhinitis, long-term safety and tolerance will be crucial for any therapy treating neurological or psychiatric diseases. All the ciliostatics listed above are preservatives, which will be necessary for the stability of some formulations and can function in both roles.

Whether mucoadhesives and ciliostatics increase intranasal brain AUC by merely prolonging nasal residence time or some other mechanism is not clear. But more time in the cavity and mucus means more interaction with the network of intracellular and extracellular xenobiotic metabolism and proteases. Furthermore, if a patient develops a hypersensitivity to the drug, this will be less well tolerated. Optimization of these formulations will be dictated by this balance of absorption and in situ degradation.

### 5.5. Biogels

Biogels are another strategy for significantly increasing nasal retention time and absorption. Biogels are defined as solutions which can modulate or tune their viscosity in response to a physical or chemical stimulus. In intranasal drug administration, this means increasing loading dose efficiency and potentiation of release times; not unlike many mucoadhesives. Numerous polymers can serve as biogels, e.g., chitosan, poloxamer, derivatives of polyacrylic acid, or cellulose. This is far from an exhaustive list, and many more examples can be found depending on which trigger is desired.

Early evidence for biogels is promising. One study found the brain AUC of intranasal rufinamide to be doubled when administered in a xyloglucan-based, heat triggered biogel, as compared to a simpler suspension [101]. This particular example is an antiepileptic which when given orally has a poor bioavailability across the BBB, demonstrating the potential of intranasal administration. Additional studies have shown improved brain AUCs for other neurologic diseases too: pluronic acid and Carbopol^®^ gels with rivastigmine for AD [102], poloxamer gels with rasagiline for PD [103], multiple gels with several drugs for treatment of depression [104,105,106], or even cellulose derivatives with paeonol for the treatment of ischemic and hemorrhagic brain injury [107]. While the clinical significance of these improved CNS bioavailabilities is unclear, biogels have a compelling and growing body of evidence suggesting that they are a promising strategy for intranasal drug administration and are worthy of studying in proper clinical trials.

### 5.6. Devices

**Figure 4:**
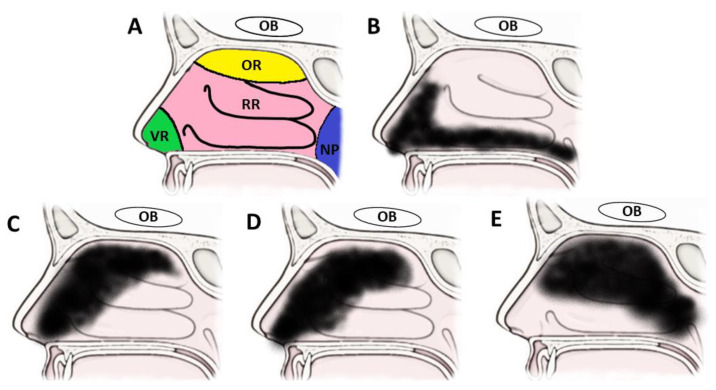
Estimation of deposition by nasal spray devices based on human in vivo studies. (**A**) regions of nasal cavity including the anterior vestibular (VR), superior olfactory (OR) and large respiratory regions (RR), as well as the posterior oropharynx (NP). The olfactory nerve is in the olfactory region and goes to the olfactory bulb (OB), while the trigeminal nerve is found in the respiratory regions and goes to the pons. (**B**) distribution of traditional nasal sprays is limited to vestibular and lower respiratory regions. (**C**–**E**) distributions of Vianase™ (**C**), Optinose Opt-Powder™ (**D**), and Impel POD^®^ (**E**) all demonstrate significantly more dose reaching the olfactory region.

Intranasal administration devices are another compelling strategy that will find a role in the clinical use of intranasal drugs. Recall that the olfactory region is <10% of the entire nasal cavity and located on the superior aspect as well as the rapid mucus clearance in the motile respiratory regions. Biology dictates that delivering the highest dose to the correct area is necessary to achieve meaningful clinical applications. Traditional spray pumps tend to only reach the anterior and lateral aspects of the nasal cavity, with <3% of the dose reaching the olfactory region [136] (Figure 4). Other alternatives such as nasal drops require the patients to precisely position their bodies, which is not suitable for many patients of all ages. The data listed so far have been conducted by trained professionals on model subjects positioned optimally in a controlled environment. The need to replicate this efficient delivery to the olfactory region in particular will be required to see results translate from labs into clinics. Fortunately, there are solutions for this challenge in the form of devices. Even these are not a magic solution. Most therapies require the patient to be conscious and cooperative with the procedure of inserting the device and triggering the release. While these devices will enable many more drugs to be clinically relevant, this approach will still pose a challenge for the very young and neurologically impaired alike.

Delivery devices all have the same goal of getting more of the dose to the olfactory region but do so by a variety of methods. While these methods will be detailed below, it is important to note that cross-comparisons are difficult as they use different formulations (liquids vs. powders) and measure different endpoints (or even different definitions of the same endpoint, such as the olfactory region itself). The advantages and disadvantages of each system provide an opportunity for optimal pairings, depending on each specific drug and disease in question.

Several types of devices have been produced and studied to date, though all in a preclinical context. First are electronic nebulizers, such as ViaNase™. This device has been used in studies to administer insulin for the treatment of AD, with significant improvement in cognition noted [108,109]. This is a great example of the untapped potential clinical improvements of the insulin administration route. These devices require additional research as there is limited evidence that they actually increase delivery to the olfactory region and do not release a substantial amount of dose into the lungs, where additional irritation or damage could occur [110]. There is a nitrogen-propelled version of this system, though these devices have yet to be studied in humans in a meaningful capacity.

Many powdered devices are available from a wide variety of pharmaceutical companies. Powdered formulations have the advantage of increased stability and in some cases improved nasal residence. One example of a powdered device designed for intranasal nose-to-brain delivery is the Opt-Powder^TM^, made by the Optinose company [136]. This device also shows another advantage of powders, as more than six times the powder was delivered to the upper nasal cavity compared to liquids. This may just be that the device is more optimized for powders, there are devices that can deliver both powders and liquids effectively, such as the Precision Olfactory Delivery (POD^®^) device from Impel NeuroPharma [111]. Both these devices are powered by the patient simultaneously exhaling from their mouth. While this forces closure of the soft palate and lessens the dose accidentally arriving in the lungs, many patients with reduced pulmonary or cognitive function may struggle to use the device properly.

We have detailed the various additives, gels, nasal coatings, and devices that can be used to increase the brain AUC of intranasal administered drugs. Many appear to be capable of helping to overcome the obstacles inherent to the nasal cavity tissue and enabling this delivery route.

### 5.7. Ultrasound and Magnetophoresis

Though greatly limited in their application by the need of highly trained professionals, some preliminary research has examined other technologies to improve nose-to-brain delivery. First is magnetophoresis, whereby the drug is attached to a magnetic particle and directed to the olfactory region of the nasal cavity to improve the dose reaching the brain [112]. These authors reported an astounding 64-fold potential increase in delivery, with nearly a 50% delivery efficiency. In select settings this approach may prove to be a massively important tool, but not in any form of repeated or self-administered use. Other researchers have looked at using focused ultrasound sonication to increase localization of a drug within the CNS. While this method did significantly improve localization, it again requires trained operators and specialized equipment [113]. Nonetheless, both strategies provide interesting potential for singular and focused treatments. These strategies are illustrative of the absolute need for a device that increases the dose reaching the olfactory region of the nasal cavity. Further replication and research will be required of these preclinical potential therapies.

## 6. Nanocarriers in Brain Drug Delivery via the Intranasal Route

There are many nanocarriers that have been evaluated for their potential use in intranasal drug delivery, including both organic and inorganic compounds. The exact strategy used will depend on the specific drug properties. Hydrophobic, large, or strongly charged molecules have particular difficulty diffusing through the mucus [132]. The same can be said for drugs which are substrates of various enzymes found along the pathway while being transported to their target tissue in the brain. While biogels and mucoadhesives share some of the roles in enhancing drug transport, nanocarriers are unique in that they function as particulates. This section will explore some of the more common and well-characterized formulations, as well as the strengths and weaknesses of each formulation.

One important limitation to all types of nanoparticles is size. Particles of 100 nm in diameter or smaller have been shown to reach the brain, while those of 900 nm in diameter appear to be too large for any delivery. This upper limit in size will be crucial to the success of any individual strategy. The charge of the nanoparticle also has a significant role on the safety and efficacy of the carrier. Positively-charged molecules are more likely to be cytotoxic and induce lysis, which could result in increased irritation and nasal damage with time. Meanwhile, negatively-charged carriers are more likely to be phagocytosed, which would be removed from the nose-to-brain pathway or are transported by the much less efficient intracellular pathway [137,138,139,140]. Inorganic molecules have also been shown to be more cytotoxic than organics, making them less appealing candidates in the intranasal setting [137,138,139].

### 6.1. Polymer Nanocarrier Formulations 

The first major class of nanocarriers are those derived from polymers. Chitosan and polymer-coupled chitosan derivatives have shown particular promise in recent years. One study found the use of chitosan nanoparticles allowed for the intranasal delivery of leucine-5-enkaphalin (LENK, an opioid receptor agonist) to the brain. When administered as a solution alone it resulted in no transport into the CNS [114]. Several other studies have replicated this result, showing as much as a five-fold increase in the delivery of drugs, e.g., rivastigmine, quetiapine, and pramipexole to the brain [115,116,117]. There is even evidence of the delivery of functioning siRNAs and plasmids to the brain using chitosan-derived nanoparticles [118,119,141]. One study using an siRNA targeting the chemotherapy-resistant gene for galectin-1 in mice demonstrated improved survival on temozolamide therapy, when given in a chitosan-tripolyphosphate carrier [142]. Not only can the nucleotides be delivered intact, but clinically significant doses appear possible even now in the early stages of research. As gene-based therapies continue to advance, this carrier has the potential to become particularly exciting in future research. Chitosan nanoparticles have successfully delivered lipid particles containing resveratrol to the CSF at six-fold greater concentrations than when the lipid particles were administered alone intranasally. No resveratrol lipid particles were transported to the CSF following IV administration [143]. For lipophilic drugs which would poorly penetrate the mucus layer otherwise, this particular evidence is exciting.

The exact mechanism by which these chitosan nanoparticles function is unknown, and will require additional research to fully elucidate. Though chitosan itself is known to be both a mucoadhesive and transiently open TJs, the nanoparticles do not always share this functionality. One derivative, *N*-palmitoyl-*N*-monomethyl-*N*,*N*-dimethyl-*N*,*N*,*N*-trimethyl-6-*O*-glycochitosan (or Nanomerics’ molecular envelope technology) has been used to successfully deliver LENK as discussed in Section 6, but is known to not affect the function of TJs [144]. While the platform has exciting early evidence of viability, fuller characterization will be needed as it translates into human clinical settings.

Another nanoparticle which is known to be safe in humans and can transport either degradation-prone or hydrophobic drugs is poly(L-lactide-co-glycolide), or PGLA. PGLA nanoparticles have been shown to improve intranasal delivery of the small molecule olanzapine 10-fold over a simple solution alone, and further studies have demonstrated that this can cause seizure reduction in epileptic rats [120,121]. Though the preliminary evidence is as promising as chitosan, the mechanism is even less well-characterized; PGLA is not a known mucoadhesive or permeability enhancer like chitosan. PGLA can be conjugated with compounds to enhance delivery to target tissues. Various chitosan derivatives and lectins or ligands specific to the nasal epithelium have been successfully added to PGLA particles and shown to increase delivery efficiency [122,123,124]. The chitosan particles were again found to work the best, and even seemed to have an effect on rate of movement within the brain. No tissue-level deposition has been studied, so this potential example of targeting requires further evaluation. Even PLA nanoparticles alone (PGLA without the polyglycolic acid monomers) have been shown to improve intranasal transport 5.6–7.7-fold over solutions alone, as seen in wheat germ agglutinin-conjugated poly (ethene glycol)-poly (lactic acid) (WGA-PEG-PLA) coated coumarin [145].

This is far from an exhaustive list, and more polymer-based nanoparticles are being studied as the carriers listed above and novel ones are better characterized and developed.

### 6.2. Lipid Nanocarrier Formulations

Lipid-based nanoparticles have come a long way from their liposomal origin and now offer several solid lipid nanoparticle formulations which have shown promise for intranasal administration. Many lipid particles have the benefits of being more stable during storage and cheaper in mass production compared to their aqueous, polymer-based counterparts [125]. The composition of lipids must be carefully controlled. Phosphatidylcholine, phosphatidylserine, and phosphatidylethylamine are all known P-gp substrates, and their inclusion would lead to rapid clearance in an untreated nasal epithelium without reaching the CNS [126].

Microemulsions have been used to increase the delivery of hydrophobic drugs to the brain, such as the acetylcholinesterase inhibitor tacrine [127]. This formulation not only increased the brain AUC of tacrine compared to IV administration, but when a mucoadhesive was added to the emulsion the brain AUC was increased further. This result for mucoadhesive microemulsions has been repeated in rodents with several other drugs, e.g., risperidone, paliperidone, and olanzapine [15]. Interestingly, this mucoadhesive property may be required, as other studies using microemulsions alone found a lower brain AUC compared to IV administration for almost all regions of the brain. While this may be specific to the studied drug, nimopidine, the current evidence indicates that microemulsions are most effective with an increased content of mucoadhesive.

Solid lipid nanoparticles (SLNs) are an increasingly exciting lipid formulation strategy. Even more stable and cheaper to manufacture than microemulsions, SLNs also offer slower release and stability as a solid (which would be superior for powdered delivery devices) [125]. Though most research on SLNs is focused on delivery of anticancer therapies, several studies have shown promise for intranasal delivery to the CNS. One such study showed a 10-fold increase in delivery of the antipsychotic risperidone when carried by SLNs compared to a simple solution [128,129,130]. Should powder-based delivery devices prove more effective than liquids or mists, this nanocarrier strategy may be of particular interest. The added stability at room temperature in solid form could reduce the chances of spoilage or contamination. As objects as large as stem cells have been successfully delivered to the brain intranasally, the risk of CNS infection is far from trivial. Newer forms of SLNs are also called nanostructured lipid carriers (NLCs) in the literature, and though they have similar properties, they have not been studied in delivery models.

## 7. Recent Patents in Intranasal Drug Delivery Systems

Since Dr Frey’s first patent in 1997, hundreds of patents for intranasal delivery ranging from drugs to nanoparticles to solvents have been filed for approval [6,146,147]. There are several excellent reviews on this subject, and to list every patent in detail here would be excessive in length; instead, this section will focus on the larger trends and popular types of patents. Of note, many FDA-approved intranasal drugs or therapies at the time of publication are actually for vaccines, which are absorbed well systemically via the rich vascular supply of the respiratory mucosa. Since these are not designed to target the brain, they will not be included in the discussion. Devices which enhance nose-to-brain administration follow a similar pattern. The Optinose™, ViaNase™, and POD^TM^ devices discussed above have been specifically evaluated for delivery to the brain, though there are many other patented devices for delivery to the nasal cavity [147,148]. Nonetheless, their existence should be acknowledged.

More than 60 different drugs have been patented for intranasal delivery. These include the synthetic drugs, peptides, and hormones listed above, as well as nucleic acids and many more signaling molecules. These drugs are targeted for the treatment of neurodegenerative diseases, psychiatric disorders, headaches/migraines, and traumatic brain injuries as well as pain, obesity, sleep disturbances, and cancers. Truly this breadth speaks to the wide potential of this still novel administration route. If even a fraction of the patents make it to market, many patients will experience a benefit.

Well over 50 patents have been approved for solvents, including both hydrophilics such as water or glycerin as well as hydrophobics such as various organic oils or hexanes. Various alcohols, ketones, and fatty acid derivatives have been approved as well [147]. There are over 100 patents alone for surfactants, solubilizers, and gelling agents to add to these solvents. The candidates most likely to reach market have been mentioned by name in literature above, but many other options still exist. A similar number of nanoparticle and lipid coating formulations have been patented. Well-evaluated candidates such as 1-palmitoyl-2-linoleoyl-3-acetyl-rac-glycerol (PLAG), chitosan, and -polyethylene glycol (PEG) are all on the list, as well as many other polymeric compounds. These are examples of well-characterized, safe, and seemingly effective nanoparticles. Further research will be required to prove the superiority of other compounds, or to raise concerns over these leading candidates. There are several phospholipid, cholesterol, or fatty acid formulations patented for emulsions or lipid coatings, though the effectiveness of these formulations is unclear in comparison to SLNs or NLCs. Numerous chelating agents have been patented too, which would sequester Ca^2+^ and increase TJ permeability.

As shown across the various studies examined here, there is no one true formulation, carrier, or method that will work for all intranasal delivery to the brain. Given the variety of potential drugs, beyond the extensive list of those already patented, this will only be proven with time. However, the vast number of solvents, nanocarriers, and co-administered compounds which have been repeatedly shown to improve delivery also show that many of these drugs have potential for development. Further evaluation will be required to optimize these specific formulations, but the hope for success is there. Neurological diseases continue to affect greater numbers of patients every day. To date, our pharmacological tools to address this problem have been lacking, chiefly due to the restrictive BBB. The intranasal pathway offers an exciting chance to alleviate a tremendous load of disease burden in patients of all ages; these formulations may enable many CNS disorders.

## 8. Clinical Evidence of Intranasal Delivery to the Brain Therapies

Few trials have evaluated intranasal delivery in humans with endpoints assessing clinical efficacy. The evidence from them currently is insufficient to judge the entire delivery pathway. Nonetheless, we shall review two notable examples of intranasally administered drugs in humans and their efficacy.

### 8.1. Oxytocin

Oxytocin is a reproductive neuropeptide associated with increasing social behavior and memory in animals. When given to healthy humans, intranasal oxytocin has been shown to increase trusting behaviors, e.g., social affiliation, altruism, and empathy [11,148,149]. The hope was these findings would benefit patients with autism spectrum disorder (ASD), which is becoming increasingly prevalent and characterized by hallmark deficiencies in these and other behaviors. The results of initial studies were promising, as the intranasal oxytocin improved symptoms such as emotional recognition and communication skills in adolescent males with ASD [150,151]. These formulations were a simple aqueous solution of synthetic oxytocin (Syntocinon^®^), administered with standard nasal sprays. Subsequent studies have failed to replicate these results when randomized control groups were added and more complex and wholistic end points were used for analysis [11,152,153].

Intranasal oxytocin is a complicated case study where the failure is likely more a reflection of the difficulties in translating results from the laboratory to the clinic than an indication the administration pathway is not viable. ASD is a very heterogenous disease, with over 100 genes involved and most unrelated to oxytocin deficiency. It is possible that oxytocin therapy would only be efficacious for certain patients, which would require genetic screening to predict efficacy. Furthermore, no trial included concurrent behavioral therapy, which is well-recognized as an essential component to the treatment plan of any patient regardless of pharmacologic interventions. Nonetheless, the evidence for intranasal oxytocin having an effect exists in the early studies. Intranasal oxytocin may still have a future role as one of many treatments for disorders such as ASD, but the current body of evidence is clear that the drug treatment will likely not work alone.

### 8.2. Insulin

Intranasal insulin is perhaps the most storied potential application of intranasal delivery to the CNS. Insulin resistance in CNS tissues has been observed in patients with AD, as well as linked to elevated levels of hyperphosphorylated tau and β-amyloid deposition (both crucial to the pathogenesis of AD) [108,109]. Insulin receptors in the brain have been well described and implicated in functions beyond simply glucose metabolism [154]. Transport of insulin into the CNS is tightly regulated and saturated [155]. Insulin concentrations of CSF are dependent on serum concentration, rising only after an increase in serum concentration and peaking 30 min later [155]. Insulin concentrations of CSF will also be lower in magnitude. This shows the potential of intranasal delivery and bypassing the serum; insulin can be administered and achieve concentrations in the CSF that would otherwise be limited by massive peripheral effects when administered parenterally. Early studies showed intranasal insulin preserved cognition and enhanced cerebral glucose metabolism in patients with AD [109]. Notably, this study used the ViaNase™ device to optimize the insulin dose reaching the olfactory region of the nasal cavity and therefore the brain. When the researchers repeated the trial, adding multiple sites and many more patients, they were unable to replicate the results and instead found no significant difference in either outcome [156]. This study again started using the ViaNase™ device but switched to the POD^®^ device early on due to repeated malfunction of the first device. Notably the sub-group patients in this study who used the same ViaNase™ device did again demonstrate the preservation of cognition after 12 months. It is possible that the ViaNase™ electric nebulization was crucial (POD^®^ is a gas-driven atomizer), but this study was not designed for device comparisons. It is also impossible to assert if the difference is due to the different device use or the small sample size. It is not an unreasonable thought, since another study analyzing only the ViaNase™ patients did show a reduction in hippocampal white matter loss, compared to the placebo group using the device without insulin [157]. However, this reanalysis is further limited as neither study directly measured CSF insulin concentrations. Despite the immediate lack of results in the first phase 2/3 clinical trial, intranasal insulin still holds significant promise as a therapy for AD. A single early trial does not negate years of evidence in animal and human subjects. Instead, it is a potential reminder that not all devices or formulations are created equal; which one is used for a given drug should be considered.

There is also new evidence for insulin specifically that many of the nanocarriers described above can improve delivery to the brain, compared to native insulin alone [158]. SLNs, PGLAs and chitosan-coated formulations of both SLNs and PGLAs were tested and found to be superior for maintaining structural stability, improving nasal absorption, and prolonging insulin release [158]. This is all while only considering native insulin: there are several long-acting insulin preparations available that should theoretically function the same in the CNS. However, more studies will be needed to demonstrate this phenomenon in vivo. There is considerable evidence for intranasal insulin treating AD [21,25,79,108,109,154,155,156,157]. This evidence also points to the importance of maximizing the dose reaching the brain. Whether optimization is achieved by devices, nanoparticles, or a combination, future studies must include these technologies. They may well be the tools that finally move intranasal administration from the laboratory to patients in need.

## 9. Expert Opinion

Intranasal delivery is an exciting technique because it will allow for therapeutic concentrations of drugs in the CNS which previously could not be achieved without prohibitory peripheral side effects via conventional administration routes. Insulin exemplifies this concept well; a therapeutic dose for brain tissue given parenterally would cause unsafe blood concentrations before enough crossed the BBB. With intranasal administration one can bypass the peripheral blood and, therefore, many adverse effects. Clinical studies in humans have found mixed results so far. However, the broad body of evidence before makes this appear more of an issue of optimization than viability. The evidence discussed above demonstrates nanocarriers increase dose fraction delivered intranasally, and dosing will ultimately decide the viability of this delivery mechanism. Other drug classes, e.g., antipsychotics, antiepileptics, and chemotherapies could benefit greatly from bypassing the periphery as well. Nanocarriers will need to be carefully selected to achieve a stable brain AUC for these drugs to be viable in the clinical setting.

Bypassing the bloodstream can also improve drugs which would otherwise be degraded before reaching the brain. An example used today is levodopa, which requires coadministration with carbidopa to prevent metabolism. Intranasal delivery can allow for entirely new classes of drugs never before possible including peptides such as GDNF and nerve growth factor (NGF), or future siRNAs for gene therapies. Bypassing blood can mean bypassing pathology too, such as neuro-protective insulin for ischemic strokes. However, even these fragile peptides may need a nanocarrier that can protect them from nasal proteases.

Intranasal delivery means rapid, noninvasive access to the brain enabling numerous novel therapies. However, this is not a panacea, and careful optimization will be needed for any of these new treatments to reach patients. A major factor will be the formulations and devices described in this article. Early clinical applications will likely take the form of intranasally administering already FDA-approved drugs such as antipsychotic, seizure medications, or even insulin. Then as formulations are optimized with known therapies, more novel drugs will become available. The potential of intranasal delivery cannot be emphasized enough; these formulations are key to realizing this route.

## 10. Conclusions

Intranasal delivery directly to the brain is supported by robust evidence in rodents and humans that has been replicated for decades with all kinds of therapies. Recent studies have focused on translating these results from the laboratory into the clinic, but with mixed results. Undoubtedly, much of this is due to the complexities of treating any multifactorial disease. However, this technique and administration route is so new that almost nothing has been done in terms of optimization and efficiency of dosing. The formulations, additives, and devices reviewed here offer the promise of bridging the gap between trained technicians in a laboratory setting and ordinary patients in the clinical world. Many studies have demonstrated the correct nanocarrier can increase the dose reaching brain tissue by orders of magnitude. After all, even the best drugs cannot work if they do not reach their target. Future studies are warranted to implement and validate these advances, as they may be the key in bringing a novel therapy to market.

## Figures and Tables

**Table 1:** Description of factors affecting intranasal delivery.

Factor	Summary	References
Mucus	Negatively charged gel reduces movement of large, charged, and nonpolar molecules	[34,35,36,37]
Enzymatic degradation	Antimicrobial and other enzymes in mucus and epithelial cells degrade the drug	[36]
Ciliary clearance	Ciliary turnover of mucus will remove slowly diffusing drugs	[38]
Tight junctions	Apical proteins greatly restrict drug movement across epithelium between cells	[39,40,41,42,43,44]
Intrinsic drug characteristics	Molecule weight over 1 kDa, polarity, strong charge can affect absorption	[45,46,47,48,49,50,51,52,53,54,55,56,57,58,59,60]
Formulation factors	pH, buffer capacity, osmolarity, and volume are important for liquids. Solubility is additionally important for powders	[61,62,63]
Vasculature and Lymphatics drainage	Vasculature of lamina propria can drain away drug before transport into the CNS	[64]

**Table 2:** Notable additives and strategies for intranasal delivery systems.

Additive or Formulation	Summary	Examples	References
Simple solutions	Simplest strategy which has shown to be possible but likely insufficient	PBS or Saline solutions	[83,84,85]
Nasal Permeability enhancers	Broad category of agents which disrupt nasal epithelia to increase absorption	Cyclodextrans, Sodium Hyaluronate, Cremophor RH40, Chitosan, Cyclopentyladenosine	[58,86,87,88,89,90]
Enzyme Modulators	Disrupt the normal function of enzymes in the epithelium	P-glycoprotein inhibitors, CYP450 inhibitors, Acetazolamide	[91,92,93,94,95,96]
Vasoconstrictors	Reducing the rich vascular supply causes less drug to be absorbed into circulation	Phenylephrine	[64]
Mucoadhesives	Increase adherence to mucus and residence time in cavity for better absorption	Chitosan, Carbopol^®^, Carboxymethylcellulose	[15,97,98,99]
Ciliostatics	Impaired ciliary movement decreases mucus clearance increasing residence time	Chlorbutol, Hydroxybenzoate, Phenylmercuric acid, Thiomersal	[100]
Biogels	Liquid that activates to gel in nasal cavity, increasing residence time and absorption	Pluronic/Carbopol gels, Cellulose derivatives/Paenol gels, Chitosan derivative gels	[101,102,103,104,105,106,107]
Devices	Devices target delivery of broader formulations to the olfactory region of the nasal cavity	ViaNase™, OptiNose™, Precision Olfactory Device^®^, Mechanical Spray bottles	[108,109,110,111]
US or Magnet guiding	Niche application of US or magnetic gradients to guide labeled drug delivery	Ultrasound and Magnetophoresis	[112,113]
Nanocarriers	Broad category of organic and inorganic nanoparticles that enhance absorption and delivery of bioactive drugs to brain	Chitosan, PGLA nanoparticles, Liposomes, Microemulsions, Solid-Lipid nanoparticles	[114,115,116,117,118,119,120,121,122,123,124,125,126,127,128,129,130]

## Data Availability

Not applicable.

## Abbreviations

AD	Alzheimer’s disease
ASD	autism spectrum disorder
AUC	area under curve
BBB	blood-brain barrier
CNS	central nervous system
CSF	cerebro-spinal fluid
IV	intravenous
LENK	leucine-5-enkaphalin
MMP	matrix metalloproteinase
NGF	nerve growth factor
NLC	nanostructured lipid carrier
OEF	olfactory ensheathing fibroblast
OSN	olfactory sensory neuron
PD	Parkinson’s disease
PEG	polyethylene glycol
PGLA	poly(l-lactide-co-glycolide)
RMS	rostral migratory stream
TJ	tight junction
